# MutaCYP: Classification of missense mutations in human cytochromes P450

**DOI:** 10.1186/1755-8794-7-47

**Published:** 2014-07-30

**Authors:** Kenneth Fechter, Aleksey Porollo

**Affiliations:** 1 Department of Biomedical Engineering, University of Cincinnati, Cincinnati, OH, USA; 2 Department of Environmental Health, University of Cincinnati, Cincinnati, OH, USA; 3 Center for Autoimmune Genomics and Etiology and Division of Biomedical Informatics, Cincinnati Children’s Hospital Medical Center, 3333 Burnet Avenue, Cincinnati, OH 45229, USA

**Keywords:** Human CYP variants, Human CYP polymorphism, Machine learning based prediction, Classification of missense mutations, Relative solvent accessibility, Evolutionary information

## Abstract

**Background:**

Cytochrome P450 monooxygenases (CYPs) represent a large and diverse family of enzymes involved in various biological processes in humans. Individual genome sequencing has revealed multiple mutations in human CYPs, and many missense mutations have been associated with variety of diseases. Since 3D structures are not resolved for most human CYPs, there is a need for a reliable sequence-based prediction that discriminates benign and disease causing mutations.

**Methods:**

A new prediction method (MutaCYP) has been developed for scoring *de novo* missense mutations to have a deleterious effect. The method utilizes only five features, all of which are sequence-based: predicted relative solvent accessibility (RSA), variance of predicted RSA among the residues in close sequence proximity, Z-score of Shannon entropy for a given position, difference in similarity scores and weighted difference in size between wild type and new amino acids. The method is based on a single neural network.

**Results:**

MutaCYP achieves MCC = 0.70, Q2 = 88.52%, Recall = 93.40% with Precision = 91.09%, and AUC = 0.909. Comparative evaluation with other existing methods indicates that MutaCYP outperforms SIFT and PolyPhen-2. Predictions by MutaCYP appear to be orthogonal to predictions by the evaluated methods. Potential issues on reliability of annotations of mutations in the existing databases are discussed.

**Conclusions:**

A new accurate method, MutaCYP, for classification of missense mutations in human CYPs is presented. The prediction model consists of only five sequence-based features, including a real-valued predicted relative solvent accessibility. The method is publicly available at http://research.cchmc.org/MutaSense/.

## Background

Cytochrome P450 monooxygenases (CYPs) are heme-thiolate enzymes that catalyze a broad range of reactions, including hydroxylation, epoxidation, dealkylation, and heteroatom oxygenation [1]. In humans, CYPs participate in various innate metabolic pathways, e.g., steroid hormone biosynthesis or fatty acid metabolism, and are also involved in biotransformation of xenobiotics, such as drugs and environmental pollutants [2]. Such considerable involvement in a wide array of biological processes (organ development, hormone signaling, etc.) requires a fine tuning of CYPs to function properly. Consequently, any imbalance in enzyme availability or its malfunction, e.g., due to a genetic mutation, may lead to a disease state in humans [3,4] or may change the susceptibility of an individual to environmental insults [5].

The human genome encodes 57 P450 genes grouped into 18 mammalian families. Despite the significant sequence diversity between CYP families, all proteins display common 3D structural elements shared with CYPs from other biological kingdoms [6-8]. Substrate specificity is determined by the size and shape of the active site cavity; by the availability of the substrate access channel and its physico-chemical characteristics; and by amino acid composition at the substrate recognition sites [9,10]. In addition to structural elements mentioned herein, missense mutations altering enzyme activity can occur at the heme-binding site, within the protein core, and at the protein interface involved in electron transfer from the redox partners, ferredoxin (FDX) and P450 oxidoreductase (POR) [11,12]. However, not all missense mutations in CYPs have functional implications and result in a disease phenotype. To this end, individual genome sequencing has revealed a considerable number of variants in CYP genes (see below), and this number is expected to grow with time. On the other hand, experimental assessment of the functional implications of all identified mutations is impractical. As a result, there is a growing demand from emerging personalized medicine for computational methods for the identification of disease causing mutations among the realm of CYP variants as well as for prediction of changes in drug metabolism by mutated CYPs.

There have been a number of attempts made to compile available information about CYPs, including their polymorphisms and activity. The Cytochrome P450 Engineering Database (CYPED) is a collection of all experimentally resolved 3D structures of CYPs retrieved from the Protein Data Bank (PDB) and categorized by CYP subfamilies [13]. CYPED also provides a tool for prediction of conserved modules in CYP structures [10]. However, no polymorphism data are available in this database, and human enzymes are underrepresented. Two databases provide information about CYP variants and their corresponding activity focused on drug metabolism: SuperCYP [14] and The Human Cytochrome P450 Allele Nomenclature Database [15]. The former database is limited to the single nucleotide polymorphism (SNP) data only and lacks information regarding clinical phenotypes. The latter database contains information about only half of the human CYPs (29 of 57 genes as of May 2013). More generalized public databases can be used as a better source of CYP variants: NCBI dbSNP [16] or UniProt humsavar [17]. These two databases can also contain associated disease phenotypes, although the annotations are lagging the recent publications.

There is a variety of methods for the prediction of functional implications of missense mutations, each of which utilizes a different heuristic (see recent reviews [18,19] as well as examples of recent methods [20-22]). However, there is no prediction method available designed specifically for analysis of missense mutations in CYPs, despite the importance of these enzymes in human health and their direct clinical relevance. At the same time, models generalized for the entire human proteome may not perform well with CYPs, as these enzymes have highly variable regions – substrate recognition sites (SRSs) – that cannot be recognized as critical functional spots by evolutionary based methods. On the other hand, a fraction of residues on the surface in CYPs are involved in the transient protein-protein interaction and electron transfer from a redox partner, but these residues may not be recognized as critical by the structure-based methods that consider mutations on the surface as less influential on a protein function. To fill this gap, we have developed a new method (MutaCYP) dedicated to the prediction of deleterious effects of missense mutations specifically in CYPs. MutaCYP combines evolutionary information and predicted structural information in 5 non-redundant sequence-based features in its prediction model. Our method was compared with two representative and commonly used methods: SIFT and PolyPhen-2. SIFT uses a prediction model based primarily on evolutionary information [23], whereas PolyPhen-2 adds protein structural data to the feature space [24]. MutaCYP outperforms both methods. At the same time, raw prediction scores by MutaCYP appear to be orthogonal to those by other evaluated methods. Hence, there is a potential to improve the accuracy of classification using a meta-predictor that combines predictions from these methods. MutaCYP is publicly available at http://research.cchmc.org/MutaSense/.

## Methods

### Datasets

The UniProt humsavar database (release 2012_10 of 31 October 2012) was used for the training and cross-validation of the prediction model. The release contained information about 562 variants in 51 human CYPs. Proteins containing only variants without disease association were excluded. The reasoning was that these proteins were most likely not yet annotated with respect to disease phenotype, and thus might introduce noise in the training as false negative instances. For example, according to UniProt, all known missense mutations in CYP1A1 are listed as benign. However, there is a solid body of evidence that some mutations in CYP1A1 can be disease causing [25,26]. Specifically, mutation I462V significantly increases catalytic activity of CYP1A1 and is associated with estrogen-related cancers and other physiological disorders [27,28]. After applying this exclusion criterion, 15 CYPs remained with 270 variants that were used to generate vectors, including 73 benign (true negative) and 197 deleterious (true positive) mutations. This training dataset was named TS270 (Additional file 1: Table S1). All excluded CYPs and their variants (36 and 292, respectively) were grouped as a separate blind set (named BS292; Additional file 1: Table S2), where association with a disease is not entirely clear, to see how predictions overlap and correlate between all validated methods.

The control dataset was derived from recently published literature on the new missense mutations identified in different CYPs with disease association. Inclusion criterion was the absence of the same mutation in the training set. A literature search yielded 30 new variants (29 deleterious and 1 neutral) for 4 human CYPs: CYP7B1 [29,30], CYP21A2 [31,32], CYP11B1 [33], and CYP27B1 [34-36]. The control dataset was named CS30 (Table 1). All datasets are available for download from the home page of the method.

**Table 1:** Human CYP variants used for the control set CS30

**UniProt ID/Gene**	**Mutation**	**Disease**	**Reference**
O75881|CYP7B1	T297A	Hereditary spastic paraplegia; Liver failure	[29,30]
	A394D	Hereditary spastic paraplegia; Liver failure	[29,30]
	R417C	Hereditary spastic paraplegia; Liver failure	[29,30]
	F470I	Hereditary spastic paraplegia; Liver failure	[29,30]
	R486C	Hereditary spastic paraplegia; Liver failure	[29,30]
P08686|CYP21A2	V139E	Congenital adrenal hyperplasia	[31]
	T295N	Congenital adrenal hyperplasia	[31]
	W302R	Congenital adrenal hyperplasia	[31]
	L353R	Congenital adrenal hyperplasia	[31]
	G375S	Congenital adrenal hyperplasia	[31]
	F404S	Congenital adrenal hyperplasia	[31]
	L446P	Congenital adrenal hyperplasia	[31]
	T450P	Congenital adrenal hyperplasia	[31]
	A265V	Neutral	[32]
P15538|CYP11B1	M88I	Congenital adrenal hyperplasia	[33]
	W116G	Congenital adrenal hyperplasia	[33]
	P159L	Congenital adrenal hyperplasia	[33]
	A165D	Congenital adrenal hyperplasia	[33]
	R366C	Congenital adrenal hyperplasia	[33]
	R384Q	Congenital adrenal hyperplasia	[33]
	T401A	Congenital adrenal hyperplasia	[33]
O15528|CYP27B1	G57V	Pseudovitamin D-deficiency rickets	[34]
	G73W	Pseudovitamin D-deficiency rickets	[34]
	L333F	Pseudovitamin D-deficiency rickets	[34]
	R432C	Pseudovitamin D-deficiency rickets	[34]
	R459C	Pseudovitamin D-deficiency rickets	[34]
	R492W	Pseudovitamin D-deficiency rickets	[34]
	G102E	Vitamin D-dependent rickets type 1	[35]
	P143L	Pseudovitamin D-deficiency rickets	[36]
	D164N	Pseudovitamin D-deficiency rickets	[36]

### Feature selection

With the goal of minimizing the feature space used for a prediction model, all explored sequence-based characteristics were evaluated using two inclusion criteria: (i) a feature displays a maximally possible discriminatory power quantified by F-score, which is defined below; (ii) a feature needs to be non-redundant to other already included features, with redundancy measured by Pearson correlation coefficient (*r*). F-score is defined as follows and was previously introduced for feature selection for prediction of protein-protein interaction sites [37].

(1) $$F=\frac{\left|{\bar{x}}_{n}\right)-\left({\bar{x}}_{d}\right|}{\sigma_{n}+\sigma_{d}}$$

where ${\bar{x}}_{n}$ and ${\bar{x}}_{d}$ are means of the feature over neutral and deleterious mutations, respectively; *σ*_*n*_ and *σ*_*d*_ are the corresponding standard deviations.

Evaluated features include physico-chemical properties of amino acids and their changes due to mutation, position specific similarity scores and Shannon entropy derived from multiple sequence alignment (MSA), predicted relative solvent accessibility (RSA). MSA was obtained using PSI-BLAST with three iterations against the NCBI nr database, following our previous protocol [38]. The complete list of the explored features along with their descriptions is available in Additional file 1: Table S3.

**Figure 1:**
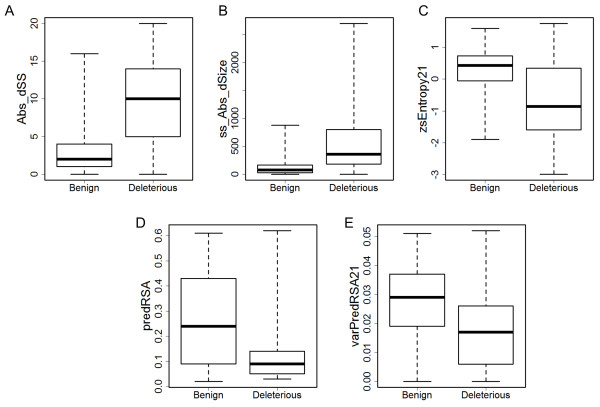
**Distribution of the features used in the final prediction model over benign and deleterious mutations. A**. Abs_dSS – absolute difference between similarity scores of wild type amino acid and mutation for a given position. **B**. ss_Abs_dSize – absolute difference between sizes of wild type amino acid and mutation weighted by the difference of the corresponding similarity scores. **C**. zsEntropy21 – Z-score for Shannon entropy at a given position based on a window of 21 neighboring amino acids. **D**. predRSA – predicted RSA. **E**. varPredRSA21 – variance of predicted RSA for the window of 21 neighboring amino acids. Whiskers indicate minimal and maximal values of a given feature.

Feature selection is summarized in Additional file 1: Table S4. The best performance was observed with the set of features that have F-score ≥ 0.4 and *r* < 0.8. The final feature space used in subsequent evaluations and in MutaCYP consists of 5 features: absolute difference between similarity scores of wild type amino acid and mutation for a given position (Abs_dSS); absolute difference between sizes of wild type amino acid and mutation weighted by the difference of the corresponding similarity scores (ss_Abs_dSize); Z-score for Shannon entropy at a given position based on a window of 21 neighboring amino acids (zsEntropy21); predicted RSA (predRSA); and variance of predicted RSA for the window of 21 neighboring amino acids (varPredRSA21). Figure 1 displays the distributions of these five features for benign and disease causing mutations; Table 2 contains the corresponding F-scores and pairwise correlation coefficients.

**Table 2:** Features passed the inclusion criteria and used for the final prediction model

**Feature**	***F-score***	**Correlation, *r***			
		**ss_Abs_dSize**	**zsEntropy21**	**predRSA**	**varPredRSA21**
Abs_dSS	0.73	0.73	−0.72	−0.38	−0.32
ss_Abs_dSize	0.61		−0.50	−0.28	−0.31
zsEntropy21	0.49			0.39	0.14
predRSA	0.47				0.42
varPredRSA21	0.45				

### Models and validation

Two models, linear and non-linear, were used in this work. The former model was based on linear discriminant analysis (LDA) as implemented in the TOOLDIAG package [39]. The LDA-based models were employed for feature selection. The non-linear model is based on neural networks (NN) as implemented in the SNNS package [40]. Multiple NN architectures were evaluated. All NNs were trained using standard backpropagation (StdBP) and resilient backpropagation (Rprop) learning algorithms. Comparative analysis of the performance of different NNs can be found in Additional file 1: Table S5. The best performing NN appeared to be a feed forward network with 5 input nodes, 2 hidden layers with 10 and 5 hidden nodes, and 2 output nodes, trained using the Rprop learning algorithm.

All models herein were evaluated using 5-fold cross-validation on the training set. In case of NN-based models, additional 20% of vectors were withdrawn from each training subset to be used as a validation subset for choosing the best performing NN in each fold, which is then evaluated using a corresponding test subset. The final model employed in MutaCYP is a single NN that showed the best generalization from the validation to test subsets (Additional file 1: Table S5). A flowchart depicting the protocol for building a prediction model used in MutaCYP can be found in supplementary materials (Additional file 2: Figure S1).

### Accuracy measures

The following measures of prediction accuracy were used in this work: the two-class classification accuracy (Q_2_), recall (R), precision (P), and Matthews correlation coefficient (MCC).

(2) $$Q_{2}=\frac{\mathrm{TP}+\mathrm{TN}}{\mathrm{TP}+\mathrm{TN}+\mathrm{FP}+\mathrm{FN}}\cdot 100\%$$

(3) $$R=\frac{\mathrm{TP}}{\mathrm{TP}+\mathrm{FN}}\cdot 100\%$$

(4) $$P=\frac{\mathrm{TP}}{\mathrm{TP}+\mathrm{FP}}\cdot 100\%$$

(5) $$\mathrm{MCC}=\frac{\mathrm{TP}\cdot \mathrm{TN}-\mathrm{FP}\cdot \mathrm{FN}}{\sqrt{\left(\mathrm{TP}+\mathrm{FN}\right)\left(\mathrm{TP}+\mathrm{FP}\right)\left(\mathrm{TN}+\mathrm{FP}\right)\left(\mathrm{TN}+\mathrm{FN}\right)}}$$

where TP are true positives (deleterious mutations), TN – true negatives (benign mutations), FP – false positives, and FN – false negatives. MCC was used as the objective function to be maximized during feature selection, and as a measure of generalization during selection of NN for the final prediction model (Additional file 1: Tables S4 and S5).

## Results and discussion

### Feature selection

Missense mutations in CYPs may cause a disease phenotype due to many reasons including impediment of heme binding; misfolding or destabilizing of the protein; a change in the binding affinity to a substrate leading to reduced or increased enzymatic activity; alteration of the substrate/product turnaround in and out of the active site cavity; hindrance or abolishment of the binding to a redox partner (affecting the electron transport rate); and an altered ability to reside at the membrane such that the protein cannot properly localize within the cell.

Structure-based features, while being the most informative, cannot be used to full advantage, as most of the human CYPs are not structurally resolved. For example, it is difficult to map SRSs using sequence homology because they are highly variable regions. Moreover, some enzymes have a well-defined substrate access channel, whereas others do not. The protein-protein interaction interface with a redox partner is also not defined for human CYPs and cannot be mapped directly from remote homologs. Hence, a fraction of missense mutations on the surface cannot be discarded from the pool of potential effectors, as some prediction methods are inclined to do. The model for the prediction method presented herein utilizes sequence-based characteristics only.

Since many considered features are evolutionary based, the influence of MSA quality on these features was explored. In addition to a full sized NCBI nr database, two reduced nr versions were used to generate MSA, where redundant sequences with 90% and 70% identity were removed. Sequence clustering was performed using CD-HIT [41]. Changes in discriminatory power (F-scores) of the evolutionary based features depending on the sequence database used are summarized in Additional file 1: Table S3. Features based on MSA derived from the nr database, reduced by removing redundant sequences with over 90% identity (nr90), appear to provide the best distinction between benign and deleterious mutations (Additional file 1: Table S3, *F*^b^ column). Hence, the following results and the final prediction model are based on the nr90 sequence database.

As expected, evolutionary-based features indicate that disease causing mutations occur in CYPs primarily at conserved sites and have unfavorable similarity scores for mutation amino acids. In this respect, the distribution of Abs_dSS displays the tendency for deleterious mutations to have a wider difference in similarity scores between the mutation and a wild type amino acid (Figure 1A, F = 0.73). Similar considerations were used as a basis for the SIFT and PolyPhen-2 methods. The former computes the probability of the occurrence of a given mutation at a given position based on MSA [23], whereas the latter method uses the dSS feature in its prediction model (see supplementary to [24]). Concordantly, ss_Abs_dSize shows a larger weighted change in size of an amino acid for deleterious mutations (Figure 1B, F = 0.61). Furthermore, Figure 1C shows that entropy for positions with deleterious mutations is shifted from the average entropy across neighboring residues towards negative direction, indicating a higher conservation (zsEntropy21, F = 0.49). Of note, entropy itself for deleterious mutations is closer to 0 than for benign mutations and has high F-score equal to 0.66. However, it is highly correlated with absolute difference between similarity scores, r(Abs_dSS, Entropy) = −0.82, and hence it was removed from the final feature space as a redundant feature (Additional file 1: Table S4).

More interestingly, predRSA appears among the strongest sequence-based characteristics capturing the disease causing mutations (F = 0.47). As follows from the name, RSA measures the extent of surface exposure (or conversely, a burial state) for a given residue in a given protein conformation normalized to a maximal possible exposure for a given kind of amino acid. The use of predicted RSA in the prediction of deleterious mutations is not entirely novel to this study. Two other studies reported prediction models that include predicted RSA [42,43]. However, those models used a 2- or 3-state definition of solvent accessibility (e.g., buried, half-buried, and exposed), which limits its applicability. In this regard, we previously developed our own method for RSA prediction (SABLE) and showed that this structural characteristic is more meaningful and useful when considered as a continuous value [38]. Furthermore, with the overall accurate prediction of RSA by SABLE, we showed that the method is prone to over-prediction in terms of burial state for residues that are located in trans-membrane regions [44], at protein-protein interaction interfaces [37], and within structurally restrained regions [45]. These are exactly the places where one would expect deleterious missense mutations most likely to occur. Therefore, a certain bias in predicted RSA towards the burial state is expected to correlate with such mutations (Figure 1D). To this end, variance in predicted RSA (varPredRSA21) is a complementary, yet orthogonal, feature to predRSA (F = 0.45, *r* = 0.42). It describes local sequence environment in terms of surface exposure and appears to be lower for positions with deleterious mutations (Figure 1E), indicating a homogeneous environment (for example, most of the neighboring residues are predicted to be buried).

### Evaluation of the model

Table 3 contains the summary of evaluations of the prediction models using the training set TS270. First, we assessed which of our models performs better, linear (LDA) or non-linear (NN). In 5-fold cross validation (Table 3; lines 1–2), the linear model appears to perform better, MCC = 0.54, compared to the NN-based model (MCC = 0.46). Second, we probed whether a consensus based model can improve the performance. Predictions by the five individual models derived from cross validation were combined using simple majority voting. NN-cons model shows the improvement over 5-fold cross validation, but both LDA-cons and NN-cons perform similarly yielding MCC = 0.53 (Table 3; lines 3–4). Next, in the efforts of simplifying the final prediction model, a single NN was chosen from cross validation that showed the best generalization from the validation to test subsets (see Additional file 1: Table S5 and Methods for details). Line 5 of Table 3 (highlighted with bold face) shows performance of the final model used for MutaCYP, which appears to be the best across all other models evaluated (MCC = 0.70).

**Table 3:** Performance of the prediction models on the training set TS270

**Model**	**MCC**	**Q**_**2**_**, %**	**R, %**	**P, %**
LDA 5-fold CV	0.54 ± 0.04	82.96 ± 3.19	94.47 ± 1.65	84.17 ± 4.96
NN 5-fold CV	0.46 ± 0.10	79.26 ± 4.12	87.24 ± 6.58	84.87 ± 2.20
LDA-cons	0.53	82.59	92.89	84.72
NN-cons	0.53	81.85	89.34	86.27
**MutaCYP**	**0.70**	**88.52**	**93.40**	**91.09**
PolyPhen2/HumVar	0.61	84.07	86.80	90.96
PolyPhen2/HumDiv	0.58	83.70	90.36	87.68
SIFT	0.49	76.33	77.70	85.71

LDA – linear model based on linear discriminant analysis.

NN – non-linear model based on neural networks.

LDA-cons and NN-cons – consensus models based on simple majority voting of 5 LDA or NN based models.

### Comparison with other methods

**Figure 2:**
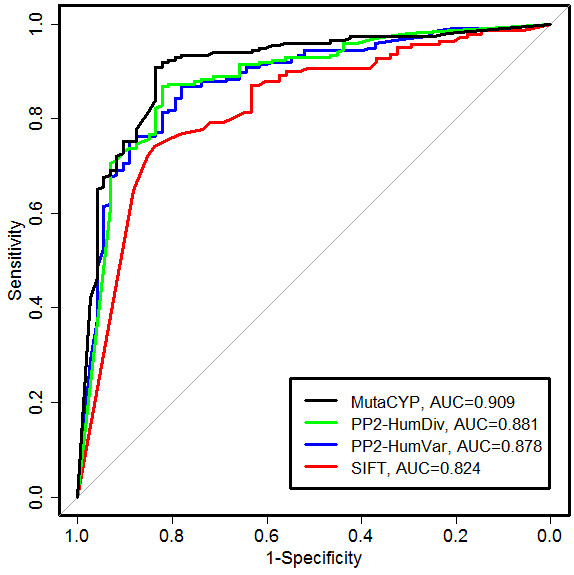
ROC curves for predictions by the evaluated methods on the TS270 dataset.

First, MutaCYP was compared with the SIFT and PolyPhen-2 methods using the TS270 set (Table 3, last three lines). Table 4 presents a confusion table for binary predictions and correlation between raw scores. Figure 2 presents ROC curves for all methods along with AUC values. Collectively, MutaCYP outperforms the other methods by all accuracy measures showing MCC = 0.70, Q2 = 88.52%, R = 93.40%, P = 91.09%, and AUC = 0.909. The SIFT method appears to perform the worst (MCC = 0.49, AUC = 0.824), and it does not provide predictions for CYP21A2 (UniProt ID: P08686; Ensembl ID: ENSP00000403721), probably due to the lack of MSA for this CYP in its pre-computed database. This excluded 63 mutations from evaluation of SIFT.

**Table 4:** Performance of the evaluated methods on the training (TS270) and control (CS30) sets

**Dataset**	**Method**^**a**^	**Confusion scores**^**b**^				**Correlation,** ***r***		
		**B-B**	**B-D**	**D-B**	**D-D**	**PolyPhen-2 HumVar**	**PolyPhen-2 HumDiv**	**SIFT**
TS270	MutaCYP	55	18	13	184	0.69	0.67	0.58
	PolyPhen-2 HumVar	56	17	26	171		0.96	0.66
	PolyPhen-2 HumDiv	48	25	19	178			0.67
	SIFT	50	18	31	108			
CS30	MutaCYP	1	0	0	29	0.38	0.19	0.12
	PolyPhen-2 HumVar	1	0	3	26		0.94	0.58
	PolyPhen-2 HumDiv	0	1	1	28			0.67
	SIFT	-	-	5	16			

^a^SIFT predictions miss 63 mutations in TS270 (58 deleterious and 5 benign) and 9 mutations in CS30 (8 deleterious and 1 benign).

^b^Confusion scores notation: B-B – the number of benign mutations predicted as benign; B-D – benign as deleterious, D-D – deleterious as deleterious; D-B – deleterious as benign.

Confusion scores are computed for binary classification. Pearson correlation coefficient is computed for real valued predictions.

Next, we compared the methods using the CS30 dataset, where 29 mutations are deleterious and not included in the UniProt humsavar database (see Methods for details). Again, SIFT did not provide prediction for CYP21A2, thus excluding 8 deleterious and 1 benign mutations from CS30. MutaCYP correctly predicted all mutations in CS30 (Table 4). PolyPhen-2 trained on the HumVar dataset correctly predicted the benign mutation (CYP21A2: A265V) and misclassified 3 deleterious mutations as benign (CYP11B1: M88I,T401A; CYP27B1:P143L). PolyPhen-2 trained on the HumDiv dataset wrongly predicted the benign mutation as deleterious, but confused only one disease causing mutation (CYP11B1:M88I). Of 21 deleterious mutations, SIFT predicted 5 mutations to be benign (CYP7B1:T297A; CYP11B1:M88I,A165D; CYP27B1:G57V,G102E), thus showing the highest confusion rate. Of note, all three compared methods predicted M88I in CYP11B1 as benign, whereas only MutaCYP correctly assigned it as deleterious.

Additional comparison of the methods has been conducted using the BS292 set (Table 5), where all mutations are assigned by UniProt as benign. Seven mutations were missing in SIFT prediction for BS292. All evaluated methods predicted considerable fraction of these mutations to be deleterious (61%, 42%, 45%, and 44% by MutaCYP, PolyPhen-2/HumVar, PolyPhen-2/HumDiv, and SIFT, respectively). These results further support our hypothesis that some missense mutations in BS292 are not fully annotated in the UniProt database. This goes in line with the previously published study, where similar concerns were raised about quality of UniProt annotations for missense mutations in cancer genes [46].

**Table 5:** Performance of the evaluated methods on the blind set (BS292)

**Method**^**a**^	**Predicted mutation status**		**Correlation,** ***r***		
	**Benign**	**Deleterious**	**PolyPhen-2 HumVar**	**PolyPhen-2 HumDiv**	**SIFT**
MutaCYP	115	177	0.48	0.48	0.34
PolyPhen-2 HumVar	170	122		0.96	0.47
PolyPhen-2 HumDiv	162	130			0.48
SIFT	161	124			

^a^SIFT predictions miss 7 mutations.

**Figure 3:**
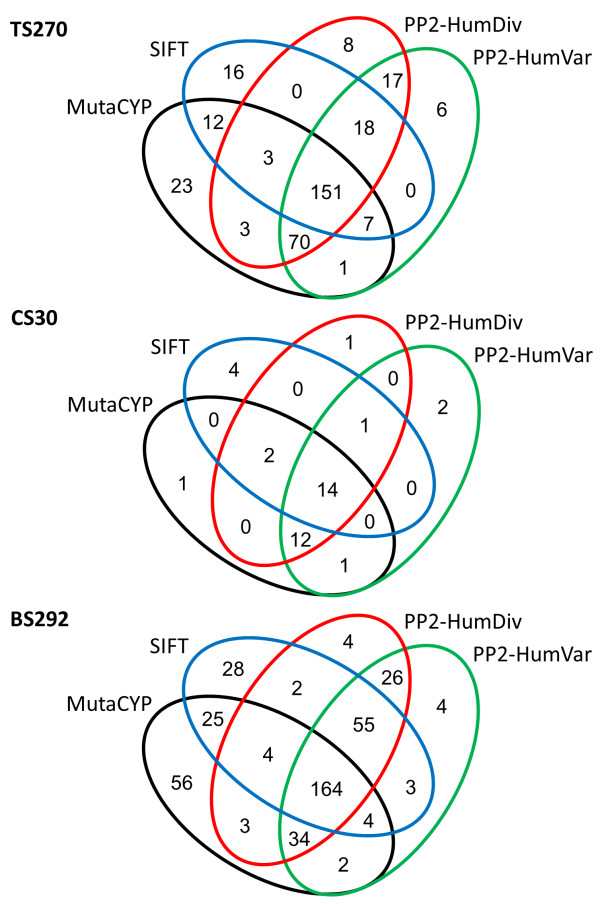
Overlap of predictions by the evaluated methods in TS270, CS30, and BS292 datasets.

Finally, we measured the correlation in predictions between the four methods by looking at the raw prediction scores (Tables 4 and 5). For TS270, MutaCYP yields moderate correlation with other methods, with Pearson correlation coefficients ranging between 0.58 and 0.69. Two PolyPhen-2 methods have the highest correlation reaching *r* = 0.96, and moderate correlation with prediction scores by SIFT (*r =* 0.66-0.67). In CS30 predictions, MutaCYP has low to moderate correlation with the other methods (*r* = 0.12-0.38). Despite the similar performance, MutaCYP has only *r* = 0.38 with PolyPhen-2 (HumVar). Again, predictions by the two PolyPhen-2 methods have the highest correlation (*r* = 0.94), and they both moderately correlate with prediction scores by SIFT (*r =* 0.58-0.67). For BS292, MutaCYP shows moderate correlation with other methods (*r* = 0.34-0.48), whereas PolyPhen-2 methods mutually correlate with *r* = 0.96, and moderately correlate with SIFT predictions (*r* = 0.47-0.48). Figure 3 illustrates the overlap between predictions by the evaluated methods using Venn diagrams. 56%, 47%, and 56% of missense mutations in the TS270, CS30, BS292 datasets, respectively, were unanimously classified by all methods.

Since MutaCYP appears orthogonal to other evaluated methods by showing only moderate correlation in raw prediction scores, there is possibility in improvement of overall prediction by combining two or more methods in a consensus-based classifier. Additional file 1: Table S6 shows the results of predictions by combinations of the considered methods using the simple majority voting and union approaches for consensus. While most of combinations do not show any improvement compared to MutaCYP alone, a consensus of MutaCYP and PolyPhen-2 trained on the HumVar data does slightly increase accuracy yielding MCC = 0.71. Perhaps, further improvement may be achieved by developing a consensus-based model using machine learning techniques, but it is beyond the scope of this study.

## Conclusions

Cytochrome P450 monooxygenases constitute a large superfamily and are represented by 57 genes in the human genome. CYPs play important roles in human health via endogenous functions and interaction with environment. A special attention is required in the analysis of missense mutations in these genes to understand their role in the disease development and individual susceptibility to environmental cues. The new method called MutaCYP was developed along with the entailing web-server to address the need in the tailored interpretation of mutations in human CYPs. With five sequence based features, MutaCYP outperforms SIFT and PolyPhen-2. Predictions by the new method appear to be orthogonal to predictions by the evaluated methods and hence can be included in a meta-predictor to further improve the accuracy of classification. The large scale analysis of missense mutations in human CYPs using 4 different prediction methods supports the notion that not all mutations in the UniProt humsavar database have reliable annotations as neutral and must be carefully used in the training and validation protocols.

## Competing interests

The authors declare that they have no competing interests.

## Authors’ contributions

KF conducted data acquisition and analysis. AP conceived of the study, implemented the prediction method, and drafted the manuscript. Both authors read and approved the final manuscript.

## Pre-publication history

The pre-publication history for this paper can be accessed here:

http://www.biomedcentral.com/1755-8794/7/47/prepub

## Supplementary Material

Additional file 1: Tables S1-S6 Mutation data from the UniProt humsavar database used for the training dataset (TS270). Table S2. Mutation data from the UniProt humsavar database used for the blind dataset (BS292). Table S3. Features considered for inclusion in the prediction model and their discriminatory power (F-score, *F*). Evolutionary based features were derived from the PSI-BLAST position specific scoring matrix (PSSM) generated after 3 iterations. Features highlighted with bold face were selected for the final model. Table S4. Performance of prediction models using features from Table S3. The accuracy in terms of MCC is based on 5-fold cross-validation of a linear model (LDA). Highlighted with bold face is the final feature space selected for MutaCYP. Table S5. Performance of neural network (NN)-based prediction models using the best feature set from Table S4. Highlighted with bold face is the final NN architecture selected for MutaCYP. Table S6. Performance of consensus-based prediction models on the training set TS270. Click here for file

Additional file 2: Figure S1 Flowchart of the protocol for developing and validating MutaCYP. The entire training dataset (TS270) was used for feature selection. A linear model (LDA) was used with 5-fold cross-validation to evaluate performance of different combinations of features. The neural network (NN) based model was used with 5-fold cross-validation to evaluate performance of different NN architectures and training algorithms. White bars represent vectors used for the training of a given model (training subset). Light grey bars represent 20% of vectors from the corresponding training subset used for choosing the best performing NN in a given training procedure (validation subset, 5f-VS in Table S5). Dark grey bars represent 20% of vectors from TS270 used for evaluation (test subset, 5f-TS in Table S5) in a given fold. A single NN that showed best accuracy on 5f-VS and generalization on 5f-TS was chosen for MutaCYP, which was subsequently evaluated using the whole training set (TS270), control set (CS30), and blind set (BS292). Click here for file
