# Supplementary material for: MutaCYP: Classification of missense mutations in human cytochromes P450
Source: BMC Med Genomics. 2014 Jul 30;7:47. doi: 10.1186/1755-8794-7-47 (PMC4119178; doi:10.1186/1755-8794-7-47)
Supplement: Additional file 1: Tables S1-S6 — Mutation data from the UniProt humsavar database used for the training dataset (TS270). Table S2. Mutation data from the UniProt humsavar database used for the blind dataset (BS292). Table S3. Features considered for inclusion in the prediction model and their discriminatory power (F-score, F). Evolutionary based features were derived from the PSI-BLAST position specific scoring matrix (PSSM) generated after 3 iterations. Features highlighted with bold face were selected for the final model. Table S4. Performance of prediction models using features from Table S3. The accuracy in terms of MCC is based on 5-fold cross-validation of a linear model (LDA). Highlighted with bold face is the final feature space selected for MutaCYP. Table S5. Performance of neural network (NN)-based prediction models using the best feature set from Table S4. Highlighted with bold face is the final NN architecture selected for MutaCYP. Table S6. Performance of consensus-based prediction models on the training set TS270. [file 1755-8794-7-47-S1.docx]

**Table 1S.** Mutation data from the UniProt humsavar database used for the training dataset (TS270).

UniProt ID|Gene Mutation dbSNP identifier Disease

P05108|CYP11A1

L141W - Adrenal insufficiency congenital with 46,XY sex reversal (AICSR) [MIM:613743]

A189V - Adrenal insufficiency congenital with 46,XY sex reversal (AICSR) [MIM:613743]

L222P - Adrenal insufficiency congenital with 46,XY sex reversal (AICSR) [MIM:613743]

E314K rs6161 -

R353W - Adrenal insufficiency congenital with 46,XY sex reversal (AICSR) [MIM:613743]

A359V - Adrenal insufficiency congenital with 46,XY sex reversal (AICSR) [MIM:613743]

V415E - Adrenal insufficiency congenital with 46,XY sex reversal (AICSR) [MIM:613743]

P15538|CYP11B1

C10Y rs6405 -

P42S - Adrenal hyperplasia type 4 (AH4) [MIM:202010]

R43Q rs4534 -

D63H rs5282 -

P94L - Adrenal hyperplasia type 4 (AH4) [MIM:202010]

N133H - Adrenal hyperplasia type 4 (AH4) [MIM:202010]

M160I rs5287 -

K173R rs4539 -

T248I rs34620645 -

F257L rs5288 -

S281N rs5291 -

L293V rs5292 -

T318M - Adrenal hyperplasia type 4 (AH4) [MIM:202010]

T318R - Adrenal hyperplasia type 4 (AH4) [MIM:202010]

T319M - Adrenal hyperplasia type 4 (AH4) [MIM:202010]

A348T rs6407 -

R374Q - Adrenal hyperplasia type 4 (AH4) [MIM:202010]

G379V - Adrenal hyperplasia type 4 (AH4) [MIM:202010]

A386V rs4541 -

R404H rs4998896 -

Y439H rs5294 -

R448H rs28934586 Adrenal hyperplasia type 4 (AH4) [MIM:202010]

R454C - Adrenal hyperplasia type 4 (AH4) [MIM:202010]

F494C - -

P19099|CYP11B2

A29T rs6438 -

R30Q rs6441 -

K173R rs4539 -

R181W rs28931609 Corticosterone methyloxidase type 2 deficiency (CMO-2 deficiency) [MIM:610600]

T185I - Corticosterone methyloxidase type 2 deficiency (CMO-2 deficiency) [MIM:610600]

E198D - Corticosterone methyloxidase type 2 deficiency (CMO-2 deficiency) [MIM:610600]

N222T rs5308 -

I248T rs4547 -

N281S rs4537 -

I339T rs4544 -

E383V rs5312 -

V386A rs4541 Corticosterone methyloxidase type 2 deficiency (CMO-2 deficiency) [MIM:610600]

V403E rs5315 -

G435S rs4545 -

L461P - Corticosterone methyloxidase type 1 deficiency (CMO-1 deficiency) [MIM:203400]

F487V rs5317 -

T498A - Corticosterone methyloxidase type 2 deficiency (CMO-2 deficiency) [MIM:610600]

P05093|CYP17A1

C22W rs762563 -

P35L - Adrenal hyperplasia type 5 (AH5) [MIM:202110]

Y64S - Adrenal hyperplasia type 5 (AH5) [MIM:202110]

F93C - Adrenal hyperplasia type 5 (AH5) [MIM:202110]

R96W - Adrenal hyperplasia type 5 (AH5) [MIM:202110]

S106P - Adrenal hyperplasia type 5 (AH5) [MIM:202110]

F114V - Adrenal hyperplasia type 5 (AH5) [MIM:202110]

D116V - Adrenal hyperplasia type 5 (AH5) [MIM:202110]

N177D - Adrenal hyperplasia type 5 (AH5) [MIM:202110]

Y329D - Adrenal hyperplasia type 5 (AH5) [MIM:202110]

P342T - Adrenal hyperplasia type 5 (AH5) [MIM:202110]

R347H - Adrenal hyperplasia type 5 (AH5) [MIM:202110]

R347C - Adrenal hyperplasia type 5 (AH5) [MIM:202110]

R358Q - Adrenal hyperplasia type 5 (AH5) [MIM:202110]

R362C - Adrenal hyperplasia type 5 (AH5) [MIM:202110]

H373L - Adrenal hyperplasia type 5 (AH5) [MIM:202110]

W406R - Adrenal hyperplasia type 5 (AH5) [MIM:202110]

F417C - Adrenal hyperplasia type 5 (AH5) [MIM:202110]

P428L - Adrenal hyperplasia type 5 (AH5) [MIM:202110]

R440H - Adrenal hyperplasia type 5 (AH5) [MIM:202110]

R496C - Adrenal hyperplasia type 5 (AH5) [MIM:202110]

R496H - Adrenal hyperplasia type 5 (AH5) [MIM:202110]

P11511|CYP19A1

W39R rs2236722 -

T201M rs28757184 -

R264C rs700519 -

R365Q - Aromatase deficiency (AROD) [MIM:613546]

R375C - Aromatase deficiency (AROD) [MIM:613546]

R375L - -

R435C - Aromatase deficiency (AROD) [MIM:613546]

C437Y - Aromatase deficiency (AROD) [MIM:613546]

Q16678|CYP1B1

S28W - Primary open angle glaucoma (POAG) [MIM:137760]

R48G rs10012 -

P52L - -

W57C - Primary open angle glaucoma (POAG) [MIM:137760]

G61E rs28936700 Primary congenital glaucoma type 3A (GLC3A) [MIM:231300]; Primary open angle glaucoma (POAG) [MIM:137760]

Q68R rs9282670 -

L77P - Primary congenital glaucoma type 3A (GLC3A) [MIM:231300]

Y81N rs9282671 Primary open angle glaucoma (POAG) [MIM:137760]

A115P - Primary congenital glaucoma type 3A (GLC3A) [MIM:231300]

A119S rs1056827 -

M132R - Primary congenital glaucoma type 3A (GLC3A) [MIM:231300]

Q144H - -

Q144P - Primary congenital glaucoma type 3A (GLC3A) [MIM:231300]

Q144R - Primary congenital glaucoma type 3A (GLC3A) [MIM:231300]

R145W - Primary open angle glaucoma (POAG) [MIM:137760]

G184S - -

D192V - Primary congenital glaucoma type 3A (GLC3A) [MIM:231300]

P193L - Primary congenital glaucoma type 3A (GLC3A) [MIM:231300]

V198I rs59472972 Primary congenital glaucoma type 3A (GLC3A) [MIM:231300]

N203S - Primary congenital glaucoma type 3A (GLC3A) [MIM:231300]

S206N rs9341248 -

S215I - Primary congenital glaucoma type 3A (GLC3A) [MIM:231300]

E229K rs57865060 Primary congenital glaucoma type 3A (GLC3A) [MIM:231300]; Primary open angle glaucoma (POAG) [MIM:137760]

G232R - Primary congenital glaucoma type 3A (GLC3A) [MIM:231300]; Primary open angle glaucoma (POAG) [MIM:137760]

S239R - Primary congenital glaucoma type 3A (GLC3A) [MIM:231300]

R266L rs9341250 -

V320L - Primary congenital glaucoma type 3A (GLC3A) [MIM:231300]

A330F - Primary congenital glaucoma type 3A (GLC3A) [MIM:231300]

L345F - Primary open angle glaucoma (POAG) [MIM:137760]

V364M - Primary congenital glaucoma type 3A (GLC3A) [MIM:231300]

G365W rs55771538 Primary congenital glaucoma type 3A (GLC3A) [MIM:231300]

R368H rs28936414 Primary congenital glaucoma type 3A (GLC3A) [MIM:231300]

D374N rs28936413 Primary congenital glaucoma type 3A (GLC3A) [MIM:231300]

P379L rs56305281 -

E387K rs55989760 Primary congenital glaucoma type 3A (GLC3A) [MIM:231300]; Primary open angle glaucoma (POAG) [MIM:137760]

A388T - Primary congenital glaucoma type 3A (GLC3A) [MIM:231300]

R390H rs56010818 Primary congenital glaucoma type 3A (GLC3A) [MIM:231300]

R390C - Primary congenital glaucoma type 3A (GLC3A) [MIM:231300]

R390S - Primary congenital glaucoma type 3A (GLC3A) [MIM:231300]

I399S - Primary congenital glaucoma type 3A (GLC3A) [MIM:231300]

V409F - Primary open angle glaucoma (POAG) [MIM:137760]

V422G - -

N423Y - Primary congenital glaucoma type 3A (GLC3A) [MIM:231300]; Primary open angle glaucoma (POAG) [MIM:137760]

L432V rs1056836 -

P437L rs56175199 Primary congenital glaucoma type 3A (GLC3A) [MIM:231300]

D441H rs4986887 -

A443G rs4986888 Primary congenital glaucoma type 3A (GLC3A) [MIM:231300]; Primary open angle glaucoma (POAG) [MIM:137760]

R444Q - Primary congenital glaucoma type 3A (GLC3A) [MIM:231300]

F445C - Primary congenital glaucoma type 3A (GLC3A) [MIM:231300]

D449E rs1056837 -

N453S rs1800440 -

G466D - Primary congenital glaucoma type 3A (GLC3A) [MIM:231300]

R469W rs28936701 Primary congenital glaucoma type 3A (GLC3A) [MIM:231300]

E499G - Primary congenital glaucoma type 3A (GLC3A) [MIM:231300]

S515L - Primary open angle glaucoma (POAG) [MIM:137760]

V518A - -

R523T - Primary open angle glaucoma (POAG) [MIM:137760]

D530G - Primary open angle glaucoma (POAG) [MIM:137760]

P08686|CYP21A2

A15T rs63749090 Adrenal hyperplasia type 3 (AH3) [MIM:201910]

P30L - Adrenal hyperplasia type 3 (AH3) [MIM:201910]

P30Q - Adrenal hyperplasia type 3 (AH3) [MIM:201910]

G56R - Adrenal hyperplasia type 3 (AH3) [MIM:201910]

H62L - Adrenal hyperplasia type 3 (AH3) [MIM:201910]

G64E - Adrenal hyperplasia type 3 (AH3) [MIM:201910]

I77T - Adrenal hyperplasia type 3 (AH3) [MIM:201910]

G90V - Adrenal hyperplasia type 3 (AH3) [MIM:201910]

K98R - -

K102R - -

P105L - Adrenal hyperplasia type 3 (AH3) [MIM:201910]

L107R - Adrenal hyperplasia type 3 (AH3) [MIM:201910]

K121Q - Adrenal hyperplasia type 3 (AH3) [MIM:201910]

R124H rs72552750 Adrenal hyperplasia type 3 (AH3) [MIM:201910]

L142P - Adrenal hyperplasia type 3 (AH3) [MIM:201910]

L167P - Adrenal hyperplasia type 3 (AH3) [MIM:201910]

C169Y - Adrenal hyperplasia type 3 (AH3) [MIM:201910]

I172N - Adrenal hyperplasia type 3 (AH3) [MIM:201910]

G178A rs72552751 Adrenal hyperplasia type 3 (AH3) [MIM:201910]

D183E rs1040310 -

V211L - Adrenal hyperplasia type 3 (AH3) [MIM:201910]

I230T - Adrenal hyperplasia type 3 (AH3) [MIM:201910]

R233K - Adrenal hyperplasia type 3 (AH3) [MIM:201910]

I236N - Adrenal hyperplasia type 3 (AH3) [MIM:201910]

V237E rs12530380 Adrenal hyperplasia type 3 (AH3) [MIM:201910]

M239K rs6476 Adrenal hyperplasia type 3 (AH3) [MIM:201910]

L261P - Adrenal hyperplasia type 3 (AH3) [MIM:201910]

S268T rs6472 -

V281L rs6471 Adrenal hyperplasia type 3 (AH3) [MIM:201910]

V281G - Adrenal hyperplasia type 3 (AH3) [MIM:201910]

M283L - Adrenal hyperplasia type 3 (AH3) [MIM:201910]

G291S - Adrenal hyperplasia type 3 (AH3) [MIM:201910]

G291R - Adrenal hyperplasia type 3 (AH3) [MIM:201910]

G291C - Adrenal hyperplasia type 3 (AH3) [MIM:201910]

G292D - Adrenal hyperplasia type 3 (AH3) [MIM:201910]

L300F - Adrenal hyperplasia type 3 (AH3) [MIM:201910]

S301Y - Adrenal hyperplasia type 3 (AH3) [MIM:201910]

L317M - Adrenal hyperplasia type 3 (AH3) [MIM:201910]

E320K - Adrenal hyperplasia type 3 (AH3) [MIM:201910]

R339H - Adrenal hyperplasia type 3 (AH3) [MIM:201910]

R341W - Adrenal hyperplasia type 3 (AH3) [MIM:201910]

R341P - Adrenal hyperplasia type 3 (AH3) [MIM:201910]

R354C - Adrenal hyperplasia type 3 (AH3) [MIM:201910]

R354H - Adrenal hyperplasia type 3 (AH3) [MIM:201910]

R356P - Adrenal hyperplasia type 3 (AH3) [MIM:201910]

R356Q - Adrenal hyperplasia type 3 (AH3) [MIM:201910]

R356W - Adrenal hyperplasia type 3 (AH3) [MIM:201910]

A362V - Adrenal hyperplasia type 3 (AH3) [MIM:201910]

L363W - Adrenal hyperplasia type 3 (AH3) [MIM:201910]

H365Y - Adrenal hyperplasia type 3 (AH3) [MIM:201910]

R369W - Adrenal hyperplasia type 3 (AH3) [MIM:201910]

E380D - Adrenal hyperplasia type 3 (AH3) [MIM:201910]

R408C - Adrenal hyperplasia type 3 (AH3) [MIM:201910]

G424S - Adrenal hyperplasia type 3 (AH3) [MIM:201910]

R426H - Adrenal hyperplasia type 3 (AH3) [MIM:201910]

R435C - Adrenal hyperplasia type 3 (AH3) [MIM:201910]

P453S rs6445 Adrenal hyperplasia type 3 (AH3) [MIM:201910]

R479L - Adrenal hyperplasia type 3 (AH3) [MIM:201910]

P482S - Adrenal hyperplasia type 3 (AH3) [MIM:201910]

R483P - Adrenal hyperplasia type 3 (AH3) [MIM:201910]

R483Q - Adrenal hyperplasia type 3 (AH3) [MIM:201910]

R483W - Adrenal hyperplasia type 3 (AH3) [MIM:201910]

N493S rs6473 -

Q07973|CYP24A1

R157Q rs35051736 -

R159Q - Hypercalcemia infantile (HCAI) [MIM:143880]

E322K - Hypercalcemia infantile (HCAI) [MIM:143880]

M374T rs6022990 -

R396W - Hypercalcemia infantile (HCAI) [MIM:143880]

L409S rs6068812 Hypercalcemia infantile (HCAI) [MIM:143880]

Q9NR63|CYP26B1

S146P - Radiohumeral fusions with other skeletal and craniofacial anomalies (RHFCA) [MIM:614416]

V181M - -

A185V - -

R191H - -

D227N - -

L264S rs2241057 -

R363L - Radiohumeral fusions with other skeletal and craniofacial anomalies (RHFCA) [MIM:614416]

E380K rs2286965 -

A420G rs7568553 -

R473C - -

V479I - -

Q02318|CYP27A1

G145E - Cerebrotendinous xanthomatosis (CTX) [MIM:213700]

A169V rs59443548 -

T175M rs2229381 -

R395C - Cerebrotendinous xanthomatosis (CTX) [MIM:213700]

R395S - Cerebrotendinous xanthomatosis (CTX) [MIM:213700]

R405Q - Cerebrotendinous xanthomatosis (CTX) [MIM:213700]

R474Q - Cerebrotendinous xanthomatosis (CTX) [MIM:213700]

R474W - Cerebrotendinous xanthomatosis (CTX) [MIM:213700]

R479C - Cerebrotendinous xanthomatosis (CTX) [MIM:213700]

O15528|CYP27B1

Q65H - Rickets vitamin D-dependent type 1A (VDDR1A) [MIM:264700]

R107H rs28934604 Rickets vitamin D-dependent type 1A (VDDR1A) [MIM:264700]

G125E rs28934605 Rickets vitamin D-dependent type 1A (VDDR1A) [MIM:264700]

V166L rs8176344 -

E189G - Rickets vitamin D-dependent type 1A (VDDR1A) [MIM:264700]

E189K - Rickets vitamin D-dependent type 1A (VDDR1A) [MIM:264700]

T321R - Rickets vitamin D-dependent type 1A (VDDR1A) [MIM:264700]

S323Y - Rickets vitamin D-dependent type 1A (VDDR1A) [MIM:264700]

R335P rs28934606 Rickets vitamin D-dependent type 1A (VDDR1A) [MIM:264700]

L343F - Rickets vitamin D-dependent type 1A (VDDR1A) [MIM:264700]

P382S rs28934607 Rickets vitamin D-dependent type 1A (VDDR1A) [MIM:264700]

R389H - Rickets vitamin D-dependent type 1A (VDDR1A) [MIM:264700]

R389G - Rickets vitamin D-dependent type 1A (VDDR1A) [MIM:264700]

R389C - Rickets vitamin D-dependent type 1A (VDDR1A) [MIM:264700]

T409I - Rickets vitamin D-dependent type 1A (VDDR1A) [MIM:264700]

R429P - Rickets vitamin D-dependent type 1A (VDDR1A) [MIM:264700]

R453C - Rickets vitamin D-dependent type 1A (VDDR1A) [MIM:264700]

V478G - Rickets vitamin D-dependent type 1A (VDDR1A) [MIM:264700]

P497R - Rickets vitamin D-dependent type 1A (VDDR1A) [MIM:264700]

Q6VVX0|CYP2R1

L99P rs61495246 Rickets vitamin D-dependent type 1B (VDDR1B) [MIM:600081]

Q6NT55|CYP4F22

F59L - Ichthyosis lamellar type 3 (LI3) [MIM:604777]

S178C rs16980531 -

R243H - Ichthyosis lamellar type 3 (LI3) [MIM:604777]

R372W - Ichthyosis lamellar type 3 (LI3) [MIM:604777]

H435Y - Ichthyosis lamellar type 3 (LI3) [MIM:604777]

H436D - Ichthyosis lamellar type 3 (LI3) [MIM:604777]

K505Q rs7256787 -

Q6ZWL3|CYP4V2

L22V rs1055138 -

W44R - Bietti crystalline corneoretinal dystrophy (BCD) [MIM:210370]

G61S - Bietti crystalline corneoretinal dystrophy (BCD) [MIM:210370]

E79D - Bietti crystalline corneoretinal dystrophy (BCD) [MIM:210370]

I111T - Bietti crystalline corneoretinal dystrophy (BCD) [MIM:210370]

M123V - Bietti crystalline corneoretinal dystrophy (BCD) [MIM:210370]

S213N rs34331648 -

Q259K rs13146272 -

E275K rs34745240 -

H331P - Bietti crystalline corneoretinal dystrophy (BCD) [MIM:210370]

S341P - Bietti crystalline corneoretinal dystrophy (BCD) [MIM:210370]

V372I - -

R443Q - -

R508H - Bietti crystalline corneoretinal dystrophy (BCD) [MIM:210370]

O75881|CYP7B1

G57R - Spastic paraplegia autosomal recessive type 5A (SPG5A) [MIM:270800]

F216S - Spastic paraplegia autosomal recessive type 5A (SPG5A) [MIM:270800]

S363F - Spastic paraplegia autosomal recessive type 5A (SPG5A) [MIM:270800]

R417H - Spastic paraplegia autosomal recessive type 5A (SPG5A) [MIM:270800]

**Table 2S.** Mutation data from the UniProt humsavar database used for the blind dataset (BS292).

UniProt ID|Gene Mutation dbSNP identifier

P04798|CYP1A1

G45D rs4646422

M66V rs35035798

I78T rs17861094

R93W rs2229150

T173R rs28399427

R279W rs34260157

I286T rs4987133

M331I rs56313657

I448N -

T461N rs1799814

I462V rs1048943

R464C -

R464S rs41279188

F470V rs36121583

R477W rs56240201

V482M rs28399429

P492R rs28399430

P05177|CYP1A2

S18C rs17861152

F21L rs56160784

P42R -

G73R rs45565238

T83M -

D104N rs34067076

L111F rs45442197

E168Q -

F186L -

F205V rs45540640

S212C -

R281W rs45468096

S298R rs17861157

G299S rs35796837

I314V rs28399418

D348N rs56276455

R377Q -

I386F -

C406Y rs55889066

R431W rs28399424

T438I rs45486893

R456H -

R457W rs34151816

Q6UW02|CYP20A1

S97L rs2043449

L346F rs1048013

Q6V0L0|CYP26C1

R245Q rs11187265

Q4G0S4|CYP27C1

T359M rs35075135

Q16696|CYP2A13

R25Q rs8192784

R101Q -

D158E -

R257C rs8192789

V323L -

F453Y -

R494C -

P11509|CYP2A6

G5R rs28399434

S29N rs28399435

V110L -

F118L rs28399440

R128Q rs4986891

R128L -

S131A rs59552350

L160H rs1801272

K194E -

R203S rs56256500

R203C -

S224P -

V292M rs2644906

T294S rs4997557

V365M rs28399454

F392Y rs1809810

N418D rs28399463

E419D rs8192730

N438Y -

I471T rs5031016

K476R rs6413474

G479V rs5031017

R485L rs28399468

P20853|CYP2A7

F61I rs10425176

C64R rs10425169

D169E rs4142867

H274R rs4079366

A301G rs2545754

R311C rs3869579

M368T rs2261144

V479G rs12460590

P20813|CYP2B6

Q21L rs34883432

R22C rs8192709

T26S rs33973337

D28G rs33980385

R29S rs33926104

R29P rs34284776

M46V rs35303484

G99E rs36060847

K139E -

R140Q rs35773040

P167A rs3826711

Q172H rs3745274

S259R rs45482602

K262R rs2279343

N289K rs34277950

T306S rs34698757

I328T rs28399499

I391N rs35979566

R487C rs3211371

P33260|CYP2C18

T385M rs2281891

P33261|CYP2C19

L17P rs55752064

I19L rs17882687

S51G -

M74T rs28399505

E92D rs17878459

W120R rs41291556

E122A rs17885179

R132Q -

R144H rs17884712

R150H rs58973490

A161P -

F168L rs28399510

P227L rs6413438

R329H -

V331I rs3758581

R410C rs17879685

R433W rs56337013

R442C -

P10632|CYP2C8

R139K rs11572080

I244V rs11572102

I264M rs1058930

I269F rs11572103

L390S -

K399R rs10509681

P11712|CYP2C9

L19I -

R144C rs1799853

R150H rs7900194

H251R rs2256871

E272G rs9332130

R335W rs28371685

Y358C rs1057909

I359L rs1057910

I359T rs56165452

D360E rs28371686

L413P rs28371687

G417D -

P489S rs9332239

P10635|CYP2D6

V11M rs769258

R26H rs28371696

R28C -

P34S rs1065852

G42R rs5030862

A85V -

L91M rs28371703

H94R rs28371704

T107I rs28371706

F120I rs1135822

E155K rs28371710

G169R -

G212E rs5030866

L231P rs17002853

A237S rs28371717

R296C rs16947

I297L -

A300G rs1058170

S311L rs1800754

H324P rs5030867

R329L rs3915951

R343G -

R365H rs1058172

I369T -

G373S rs2856959

E410K -

E418K -

P469A rs1135833

H478Y rs28371735

S486T rs1135840

P05181|CYP2E1

R76H -

V179I rs6413419

N219D rs41299426

S366C rs41299434

V389I rs55897648

H457L rs28969387

P24903|CYP2F1

S38P rs58285195

R98P rs57670668

D218N -

Q266H -

L391P -

P490L rs7246981

P51589|CYP2J2

R49S rs11572190

V113M rs11572242

N124S rs2228113

T143A rs55753213

R158C rs56307989

I192N -

D342N rs56053398

N404Y -

Q96SQ9|CYP2S1

P466L rs34971233

Q8TAV3|CYP2W1

A181T rs3735684

Q9NYL5|CYP39A1

R23P rs12192544

R103H rs2277119

Y288H rs17856332

N324K rs7761731

P08684|CYP3A4

L15P rs12721634

G56D rs56324128

K96E rs3091339

I118V rs55951658

R130Q -

R162Q rs4986907

V170I -

D174H -

T185S rs12721627

F189S rs4987161

P218R rs55901263

S222P rs55785340

S252A rs3208363

L293P rs28371759

T349N rs10250778

T363M -

L373F rs12721629

P416L rs4986909

I431T rs1041988

M445T rs4986910

P467S rs4986913

Q9HB55|CYP3A43

T27A rs45558032

M145I rs45450092

M275I rs45621431

P340A rs680055

P20815|CYP3A5

R28C rs55817950

H30Y rs28383468

Q200R rs56411402

D277E rs28383477

A337T rs28383479

I371V rs28365092

T398N rs28365083

F446S rs41279854

I488T rs28365085

P24462|CYP3A7

V71A rs45580339

R409T rs2257401

Q02928|CYP4A11

N226S rs12759923

S353G -

F434S rs1126742

Q5TCH4|CYP4A22

R11C -

Y104F rs61507155

K121R rs2758717

R126W rs12564525

G130S rs2056900

N152Y rs2056899

V185F -

S226N rs35202523

C230S rs35156123

C231R rs10789501

K276T -

L428P rs2405599

M491I rs2758714

L509F rs4926600

P13584|CYP4B1

A111V rs45559437

R173W rs4646487

R264W rs45446505

R274Q rs45578838

S322G rs45467195

Y329S rs12094024

M331I rs2297810

R340C rs4646491

V345I -

F354C rs17102592

R375C rs2297809

R482Q rs45622937

Q9HBI6|CYP4F11

R146C rs57519667

C276R rs8104361

D284N rs1060463

Q9HCS2|CYP4F12

P13L rs16995376

T16M rs16995378

N76D rs609636

I90V rs609290

C188R rs2285888

S522G rs593818

P78329|CYP4F2

S7Y rs3093104

W12G rs3093105

G185V rs3093153

A269D rs1805040

V433M rs2108622

L519M rs3093200

Q08477|CYP4F3

H96Q rs34923393

Y106C rs35888783

A269D rs1805040

V270I rs28371536

I271T rs28371479

P98187|CYP4F8

Y125F rs2072600

P447Q rs2056822

Q86W10|CYP4Z1

P393L rs28463559

Q16850|CYP51A1

V13A rs2229188

P22680|CYP7A1

H86N rs62621283

F100S -

N233S rs8192874

D347N rs8192875

Q9UNU6|CYP8B1

S88P rs9865715

R234H -

K238R rs35764459

L357F rs35637877

**Table 3S.** Features considered for inclusion in the prediction model and their discriminatory power (F-score, *F*). Evolutionary based features were derived from the PSI-BLAST position specific scoring matrix (PSSM) generated after 3 iterations. Features highlighted with bold face were selected for the final model.

| **Acronym** | ***F*** ^a^ | ***F*** ^b^ | ***F*** ^c^ | **Description** ^d^ |
| --- | --- | --- | --- | --- |
| dSS | 0.64 | 0.68 | 0.65 | Difference between similarity scores of wild type amino acid and mutation for a given position |
| **Abs_dSS** | 0.68 | 0.73 | 0.67 | Absolute difference between similarity scores of wild type amino acid and mutation for a given position |
| Entropy | 0.63 | 0.66 | 0.63 | Shannon entropy for a given position |
| EntropyRel | 0.58 | 0.57 | 0.57 | Shannon entropy for a given position relative to other positions computed similarly to the ConSurf procedure |
| zsEntropy7 | 0.35 | 0.41 | 0.36 | Z-score for Shannon entropy at a given position based on a window of 7 neighboring amino acids |
| zsEntropy11 | 0.40 | 0.44 | 0.41 | Z-score for Shannon entropy at a given position based on a window of 11 neighboring amino acids |
| zsEntropy15 | 0.44 | 0.46 | 0.45 | Z-score for Shannon entropy at a given position based on a window of 15 neighboring amino acids |
| **zsEntropy21** | 0.48 | 0.49 | 0.49 | Z-score for Shannon entropy at a given position based on a window of 21 neighboring amino acids |
| varEntropy7 | 0.36 | 0.22 | 0.35 | Variance of Shannon entropy for the window of 7 neighboring amino acids |
| varEntropy11 | 0.39 | 0.22 | 0.35 | Variance of Shannon entropy for the window of 11 neighboring amino acids |
| varEntropy15 | 0.31 | 0.14 | 0.27 | Variance of Shannon entropy for the window of 15 neighboring amino acids |
| varEntropy21 | 0.29 | 0.16 | 0.24 | Variance of Shannon entropy for the window of 21 neighboring amino acids |
| zsPredRSA7 | 0.15 | 0.15 | 0.15 | Z-score for predicted relative solvent accessibility at a given position based on a window of 7 neighboring amino acids |
| zsPredRSA11 | 0.16 | 0.16 | 0.16 | Z-score for predicted relative solvent accessibility at a given position based on a window of 11 neighboring amino acids |
| zsPredRSA15 | 0.17 | 0.17 | 0.17 | Z-score for predicted relative solvent accessibility at a given position based on a window of 15 neighboring amino acids |
| zsPredRSA21 | 0.22 | 0.22 | 0.22 | Z-score for predicted relative solvent accessibility at a given position based on a window of 21 neighboring amino acids |
| varPredRSA7 | 0.29 | 0.29 | 0.29 | Variance of predicted relative solvent accessibility for the window of 7 neighboring amino acids |
| varPredRSA11 | 0.37 | 0.37 | 0.37 | Variance of predicted relative solvent accessibility for the window of 11 neighboring amino acids |
| varPredRSA15 | 0.40 | 0.40 | 0.40 | Variance of predicted relative solvent accessibility for the window of 15 neighboring amino acids |
| **varPredRSA21** | 0.45 | 0.45 | 0.45 | Variance of predicted relative solvent accessibility for the window of 21 neighboring amino acids |
| SSref | 0.45 | 0.51 | 0.45 | Similarity score of wild type amino acid for a given position |
| SSsnp | 0.59 | 0.60 | 0.61 | Similarity score of mutation for a given position |
| dpAA | 0.55 | 0.58 | 0.54 | Difference between probabilities of wild type amino acid and mutation for a given position |
| Abs_dpAA | 0.58 | 0.62 | 0.57 | Absolute difference between probabilities of wild type amino acid and mutation for a given position |
| pAAref | 0.52 | 0.57 | 0.51 | Probability of wild type amino acid for a given position |
| pAAsnp | 0.32 | 0.23 | 0.32 | Probability of mutation amino acid for a given position |
| ss_Abs_dHP | 0.34 | 0.39 | 0.35 | Absolute difference between hydropathy indexes of wild type amino acid and mutation for a given position weighted by the difference of the corresponding similarity scores |
| p_Abs_dHP | 0.33 | 0.36 | 0.33 | Absolute difference between hydropathy indexes of wild type amino acid and mutation for a given position weighted by the difference of the corresponding probabilities |
| **ss_Abs_dSize** | 0.56 | 0.61 | 0.54 | Absolute difference between sizes of wild type amino acid and mutation for a given position weighted by the difference of the corresponding similarity scores |
| p_Abs_dSize | 0.54 | 0.58 | 0.53 | Absolute difference between sizes of wild type amino acid and mutation for a given position weighted by the difference of the corresponding probabilities |
| **PredRSA** | 0.47 | 0.47 | 0.47 | Relative solvent accessibility predicted by SABLE |
| PredTM | 0.02 | 0.02 | 0.02 | Binary value indicating whether a given position is at the predicted transmembrane region |
| HPref | 0.08 | 0.08 | 0.08 | Kyte-Doolittle hydropathy index for the wild type amino acid at a given position |
| HPsnp | 0.04 | 0.04 | 0.04 | Kyte-Doolittle hydropathy index for the new amino acid at a given position |
| dHP | 0.04 | 0.04 | 0.04 | Difference between hydropathy indexes of wild type amino acid and mutation for a given position |
| Abs_dHP | 0.17 | 0.17 | 0.17 | Absolute difference between hydropathy indexes of wild type amino acid and mutation for a given position |
| Size_ref | 0.08 | 0.08 | 0.08 | Size of the wild type amino acid at a given position |
| Size_snp | 0.08 | 0.08 | 0.08 | Size of the new amino acid at a given position |
| dSize | 0.00 | 0.00 | 0.00 | Difference between sizes of wild type amino acid and mutation for a given position |
| Abs_dSize | 0.24 | 0.24 | 0.24 | Absolute difference between sizes of wild type amino acid and mutation for a given position |
| RSA | 0.35 | 0.35 | 0.35 | 3D structure based relative solvent accessibility computed by DSSP for a given position |
| Func_Cavity | 0.27 | 0.27 | 0.27 | Probability of the deleterious mutation of the amino acid residue known to be within the active site cavity |
| Func_Heme | 0.28 | 0.28 | 0.28 | Probability of the deleterious mutation of the amino acid residue known to be in contact with heme |
| Func_None | 0.27 | 0.27 | 0.27 | Probability of the deleterious mutation of the amino acid residue known to be outside the active site cavity |
| pPredPPI | 0.08 | 0.08 | 0.08 | Probability of being at a protein-protein interaction interface predicted by SPPIDER |
| Abs_Pred_dRSA | 0.07 | 0.07 | 0.07 | Absolute difference between predicted relative solvent accessibility and computed from 3D structure at a given position |

^a^ PSSM is based on the NCBI nr database used in SABLE predictions.

^b^ PSSM is based on the reduced NCBI nr database after removing sequences with over 90% identity.

^c^ PSSM is based on the reduced NCBI nr database after removing sequences with over 70% identity.

^d^ Similarity scores are position specific scores derived from multiple sequence alignment (MSA) using, in this case, PSI-BLAST. They reflect likelihood of occurrence of a given amino acid at a given position based on a given sequence database used to generate MSA. Shannon entropy reflects variability of amino acids at a given position. Relative solvent accessibility measures solvent exposure of a residue in a given protein conformation normalized to a maximal solvent accessibility for a given type of amino acid.

**Table 4S.** Performance of prediction models using features from Table 3S. The accuracy in terms of MCC is based on 5-fold cross-validation of a linear model (LDA). Highlighted with bold face is the final feature space selected for MutaCYP.

| **Filter** | **Number of features in the model** | **MCC±SD** |
| --- | --- | --- |
| None | 46 | 0.40±0.09 |
| F-score ≥ 0.1 | 37 | 0.48±0.09 |
| F-score ≥ 0.2 | 31 | 0.48±0.03 |
| F-score ≥ 0.3 | 22 | 0.47±0.10 |
| F-score ≥ 0.4 | 18 | 0.50±0.09 |
| F-score ≥ 0.5 | 11 | 0.46±0.06 |
| F-score ≥ 0.6 | 6 | 0.40±0.15 |
|  |  |  |
| F-score ≥ 0.4 and r < 0.9 | 9 | 0.51±0.10 |
| **F-score ≥ 0.4 and r < 0.8** | **5** | **0.54±0.04** |
| F-score ≥ 0.4 and r < 0.7 | 3 | 0.48±0.16 |

**Table 5S.** Performance of neural network (NN)-based prediction models using the best feature set from Table 4S. Highlighted with bold face is the final NN architecture selected for MutaCYP.

| **NN architecture ^a^** | **NN learning algorithm ^b^** | **MCC(5f-VS) ^c^** | **MCC(5f-TS) ^d^** | **MCC±SD ^e^** |
| --- | --- | --- | --- | --- |
| **5-[10-5]-2** | **Rprop** | 0.53  0.61  0.71  0.49  **0.65** | 0.30  0.40  0.46  0.55  **0.58** | 0.46±0.10 |
| 5-[10-5]-2 | StdBP | 0.53  0.67  0.56  0.51  0.61 | 0.42  0.46  0.36  0.64  0.58 | 0.49±0.10 |
| 5-[5-3]-2 | Rprop | 0.46  0.61  0.56  0.55  0.61 | 0.26  0.34  0.39  0.55  0.58 | 0.42±0.12 |
| 5-[5-3]-2 | StdBP | 0.50  0.67  0.56  0.55  0.61 | 0.36  0.40  0.36  0.66  0.58 | 0.47±0.12 |
| 5-[10]-2 | Rprop | 0.41  0.61  0.61  0.44  0.58 | 0.39  0.34  0.48  0.75  0.54 | 0.50±0.14 |
| 5-[10]-2 | StdBP | 0.46  0.67  0.74  0.51  0.61 | 0.43  0.40  0.43  0.64  0.58 | 0.50±0.10 |
| 5-[5]-2 | Rprop | 0.53  0.61  0.61  0.49  0.61 | 0.29  0.52  0.40  0.72  0.58 | 0.50±0.15 |
| 5-[5]-2 | StdBP | 0.49  0.67  0.61  0.44  0.65 | 0.35  0.58  0.39  0.70  0.45 | 0.49±0.13 |
| 5-[3]-2 | Rprop | 0.49  0.67  0.56  0.47  0.58 | 0.31  0.46  0.46  0.64  0.58 | 0.49±0.11 |
| 5-[3]-2 | StdBP | 0.49  0.61  0.56  0.49  0.61 | 0.36  0.40  0.50  0.70  0.58 | 0.51±0.12 |
| 5-[2]-2 | Rprop | 0.41  0.61  0.45  0.43  0.61 | 0.39  0.34  0.40  0.57  0.58 | 0.46±0.10 |
| 5-[2]-2 | StdBP | 0.46  0.67  0.56  0.51  0.61 | 0.36  0.40  0.39  0.70  0.58 | 0.49±0.13 |
| 5-2 | Rprop | 0.49  0.67  0.25  0.47  0.42 | 0.36  0.52  0.48  0.67  0.63 | 0.53±0.11 |
| 5-2 | StdBP | 0.41  0.67  0.38  0.49  0.61 | 0.30  0.58  0.50  0.66  0.58 | 0.52±0.12 |

^a^ Numbers represent the number of nodes in a given layer. The first number is an input layer, the last number is the output layer, and the numbers in square brackets are nodes in the hidden layer(s).

^b^ Rprop – resilient backpropagation; StdBP – standard backpropagation learning algorithms.

^c^ Based on a validation subset for each of 5 folds (see section Methods for details).

^d^ Based on a test subset for each of 5 folds (see section Methods for details).

^e^ Based on 5-fold cross-validation (values from column ^d^).

**Table 6S.** Performance of consensus-based prediction models on the training set TS270.

| Methods^a^ | Consensus^b^ | Number of vectors^c^ | MCC |
| --- | --- | --- | --- |
| MutaCYP + PP2(HumVar) + PP2(HumDiv) + SIFT | SMV | 207 | 0.65 |
| MutaCYP + PP2(HumVar) + PP2(HumDiv) | SMV | 270 | 0.64 |
| MutaCYP + PP2(HumVar) + SIFT | SMV | 207 | 0.66 |
| MutaCYP + PP2(HumDiv) + SIFT | SMV | 207 | 0.65 |
| MutaCYP + PP2(HumVar) | SMV | 270 | 0.71 |
| MutaCYP + PP2(HumDiv) | SMV | 270 | 0.64 |
| MutaCYP + SIFT | SMV | 207 | 0.65 |
| PP2(HumVar) + PP2(HumDiv) + SIFT | SMV | 207 | 0.63 |
| PP2(HumVar) + PP2(HumDiv) | SMV | 270 | 0.57 |
| PP2(HumVar) + SIFT | SMV | 207 | 0.62 |
| PP2(HumDiv) + SIFT | SMV | 207 | 0.56 |
| MutaCYP + PP2(HumVar) + PP2(HumDiv) + SIFT | Union | 207 | 0.61 |
| MutaCYP + PP2(HumVar) + PP2(HumDiv) | Union | 270 | 0.64 |
| MutaCYP + PP2(HumVar) + SIFT | Union | 207 | 0.67 |
| MutaCYP + PP2(HumDiv) + SIFT | Union | 207 | 0.61 |
| MutaCYP + PP2(HumVar) | Union | 270 | 0.71 |
| MutaCYP + PP2(HumDiv) | Union | 270 | 0.64 |
| MutaCYP + SIFT | Union | 207 | 0.65 |
| PP2(HumVar) + PP2(HumDiv) + SIFT | Union | 207 | 0.56 |
| PP2(HumVar) + PP2(HumDiv) | Union | 270 | 0.57 |
| PP2(HumVar) + SIFT | Union | 207 | 0.62 |
| PP2(HumDiv) + SIFT | Union | 207 | 0.56 |

^a^PP2(HumVar) and PP2(HumDiv) – PolyPhen-2 trained on HumVar and HumDiv data, respectively.

^b^SMV – simple majority voting; for consensuses with the even number of methods the even vote was in favor of the deleterious class.

^c^SIFT predictions miss 63 mutations in TS270, hence the reduced set for evaluation of a consensus model containing SIFT.
