# Supplementary material for: MutaCYP: Classification of missense mutations in human cytochromes P450
Source: BMC Med Genomics. 2014 Jul 30;7:47. doi: 10.1186/1755-8794-7-47 (PMC4119178; doi:10.1186/1755-8794-7-47)
Supplement: Additional file 2: Figure S1 — Flowchart of the protocol for developing and validating MutaCYP. The entire training dataset (TS270) was used for feature selection. A linear model (LDA) was used with 5-fold cross-validation to evaluate performance of different combinations of features. The neural network (NN) based model was used with 5-fold cross-validation to evaluate performance of different NN architectures and training algorithms. White bars represent vectors used for the training of a given model (training subset). Light grey bars represent 20% of vectors from the corresponding training subset used for choosing the best performing NN in a given training procedure (validation subset, 5f-VS in Table S5). Dark grey bars represent 20% of vectors from TS270 used for evaluation (test subset, 5f-TS in Table S5) in a given fold. A single NN that showed best accuracy on 5f-VS and generalization on 5f-TS was chosen for MutaCYP, which was subsequently evaluated using the whole training set (TS270), control set (CS30), and blind set (BS292). [file 1755-8794-7-47-S2.docx]

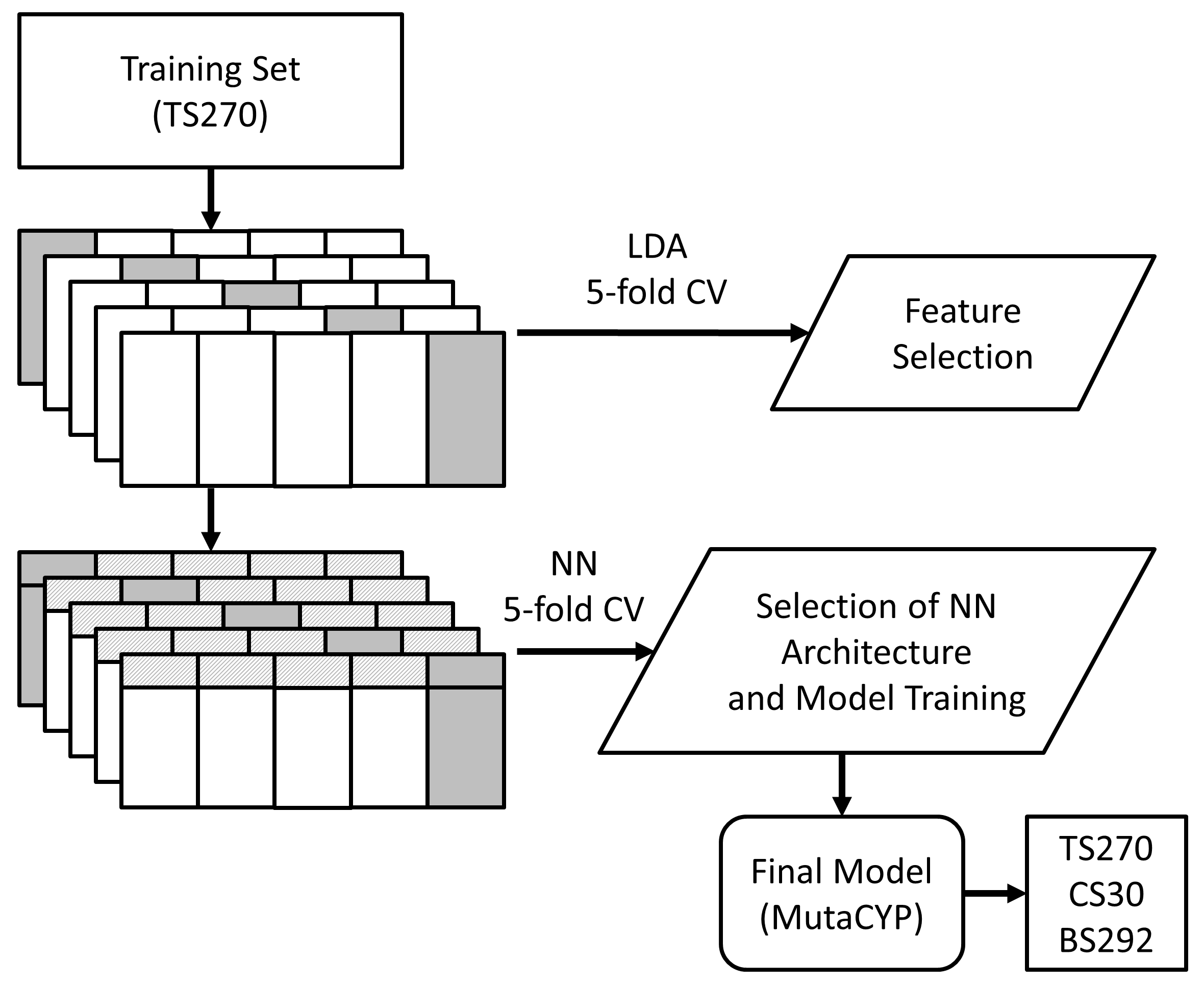


**Figure 1S.** Flowchart of the protocol for developing and validating MutaCYP. The entire training dataset (TS270) was used for feature selection. A linear model (LDA) was used with 5-fold cross-validation to evaluate performance of different combinations of features. The neural network (NN) based model was used with 5-fold cross-validation to evaluate performance of different NN architectures and training algorithms. White bars represent vectors used for the training of a given model (training subset). Light grey bars represent 20% of vectors from the corresponding training subset used for choosing the best performing NN in a given training procedure (validation subset, 5f-VS in Table 5S). Dark grey bars represent 20% of vectors from TS270 used for evaluation (test subset, 5f-TS in Table 5S) in a given fold. A single NN that showed best accuracy on 5f-VS and generalization on 5f-TS was chosen for MutaCYP, which was subsequently evaluated using the whole training set (TS270), control set (CS30), and blind set (BS292).
